# 1,1′-(Ethane-1,2-di­yl)dipyridinium bis­(1,2-di­cyano­ethene-1,2-di­thiol­ato-κ^2^ *S*,*S*′)cuprate(II)

**DOI:** 10.1107/S1600536813012439

**Published:** 2013-05-11

**Authors:** Bing-Xiang Hu, Chang-Xiao Zhou, Yang-Mei Liu, Li-Zhuang Chen, Fang-Ming Wang

**Affiliations:** a School of Biology and Chemistry Engineering, Jiangsu University of Science and Technology, Zhenjiang, Jiangsu 212003, People’s Republic of China; b National Food Packaging Products Quality Supervision and Inspection Center, Jiangsu Provincial Supervising and Testing Research Institute for Products Quality, Nanjing 210007, People’s Republic of China

## Abstract

In the title ion-pair complex, (C_12_H_14_N_2_)[Cu(C_4_N_2_S_2_)_2_], the complex anion exhibits a highly twisted coordination environment around the tetra­coordinated Cu^II^ atom. The dihedral angles between the 1,2-di­cyano­ethene-1,2-di­thiol­ato ligands and between the two pyridine rings in the cation are 37.49 (3) and 29.18 (10)°, respectively. Weak C—H⋯N and C—H⋯S hydrogen bonds link the cations and anions into a three-dimensional network.

## Related literature

For background to crystalline mol­ecular materials and coordination polymer networks, see: Brammer (2004 ▶); Robin & Fromm (2006 ▶). For 1,2-di­thiol­ene–metal complexes, see: Duan *et al.* (2010 ▶); Ni *et al.* (2005 ▶). For related structures, see: Ren *et al.* (2006 ▶); Wang *et al.* (2012 ▶).
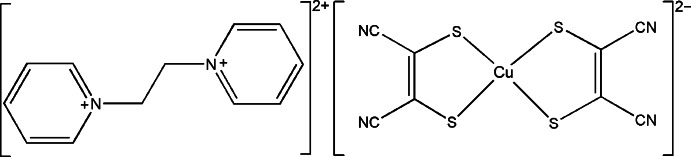


## Experimental

### 

#### Crystal data

(C_12_H_14_N_2_)[Cu(C_4_N_2_S_2_)_2_]
*M* *_r_* = 530.20
Triclinic, 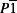

*a* = 7.7598 (10) Å
*b* = 12.3811 (15) Å
*c* = 12.6572 (16) Å
α = 77.676 (2)°
β = 72.791 (2)°
γ = 84.122 (2)°
*V* = 1133.8 (2) Å^3^
*Z* = 2
Mo *K*α radiation
μ = 1.35 mm^−1^
*T* = 291 K
0.25 × 0.20 × 0.15 mm

#### Data collection

Bruker APEX CCD diffractometer
Absorption correction: multi-scan (*SADABS*; Bruker, 2001 ▶) *T*_min_ = 0.730, *T*_max_ = 0.815
5682 measured reflections
3921 independent reflections
3217 reflections with *I* > 2σ(*I*)
*R*_int_ = 0.054

#### Refinement

*R*[*F* ^2^ > 2σ(*F* ^2^)] = 0.036
*wR*(*F* ^2^) = 0.083
*S* = 1.00
3921 reflections
280 parameters
1 restraint
H-atom parameters constrained
Δρ_max_ = 0.39 e Å^−3^
Δρ_min_ = −0.26 e Å^−3^

### 

Data collection: *SMART* (Bruker, 2007 ▶); cell refinement: *SAINT* (Bruker, 2007 ▶); data reduction: *SAINT*; program(s) used to solve structure: *SHELXTL* (Sheldrick, 2008 ▶); program(s) used to refine structure: *SHELXTL*; molecular graphics: *SHELXTL*; software used to prepare material for publication: *SHELXTL*.

## Supplementary Material

Crystal structure: contains datablock(s) I, global. DOI: 10.1107/S1600536813012439/hy2624sup1.cif

Structure factors: contains datablock(s) I. DOI: 10.1107/S1600536813012439/hy2624Isup2.hkl

Additional supplementary materials:  crystallographic information; 3D view; checkCIF report

## Figures and Tables

**Table 1:** Hydrogen-bond geometry (Å, °)

*D*—H⋯*A*	*D*—H	H⋯*A*	*D*⋯*A*	*D*—H⋯*A*
C11—H11*A*⋯N2^i^	0.93	2.61	3.356 (4)	137
C14—H14*A*⋯N4^ii^	0.97	2.60	3.341 (4)	133
C14—H14*B*⋯N1^iii^	0.97	2.60	3.479 (4)	151
C15—H15*B*⋯S1	0.97	2.83	3.769 (3)	163
C16—H16*A*⋯N2^iv^	0.93	2.56	3.368 (4)	146
C17—H17*A*⋯S2^iv^	0.93	2.82	3.743 (3)	171
C19—H19*A*⋯N3^v^	0.93	2.56	3.235 (4)	129
C20—H20*A*⋯N1^iii^	0.93	2.52	3.422 (4)	164

Symmetry codes: (i) 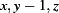; (ii) 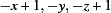; (iii) 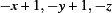; (iv) 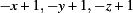; (v) 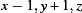.

**Fig. 1.**
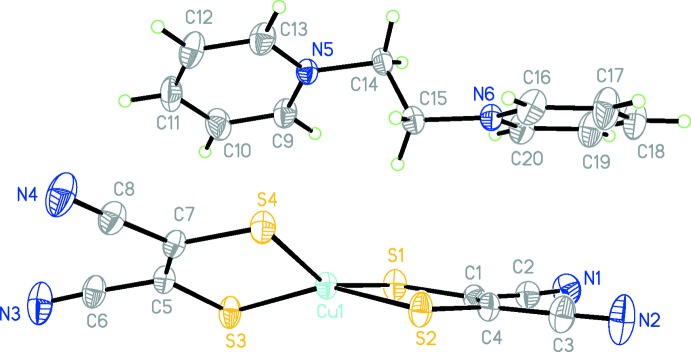
The molecular structure of the title compound. Displacement ellipsoids are drawn at the 30% probability level.

**Fig. 2.**
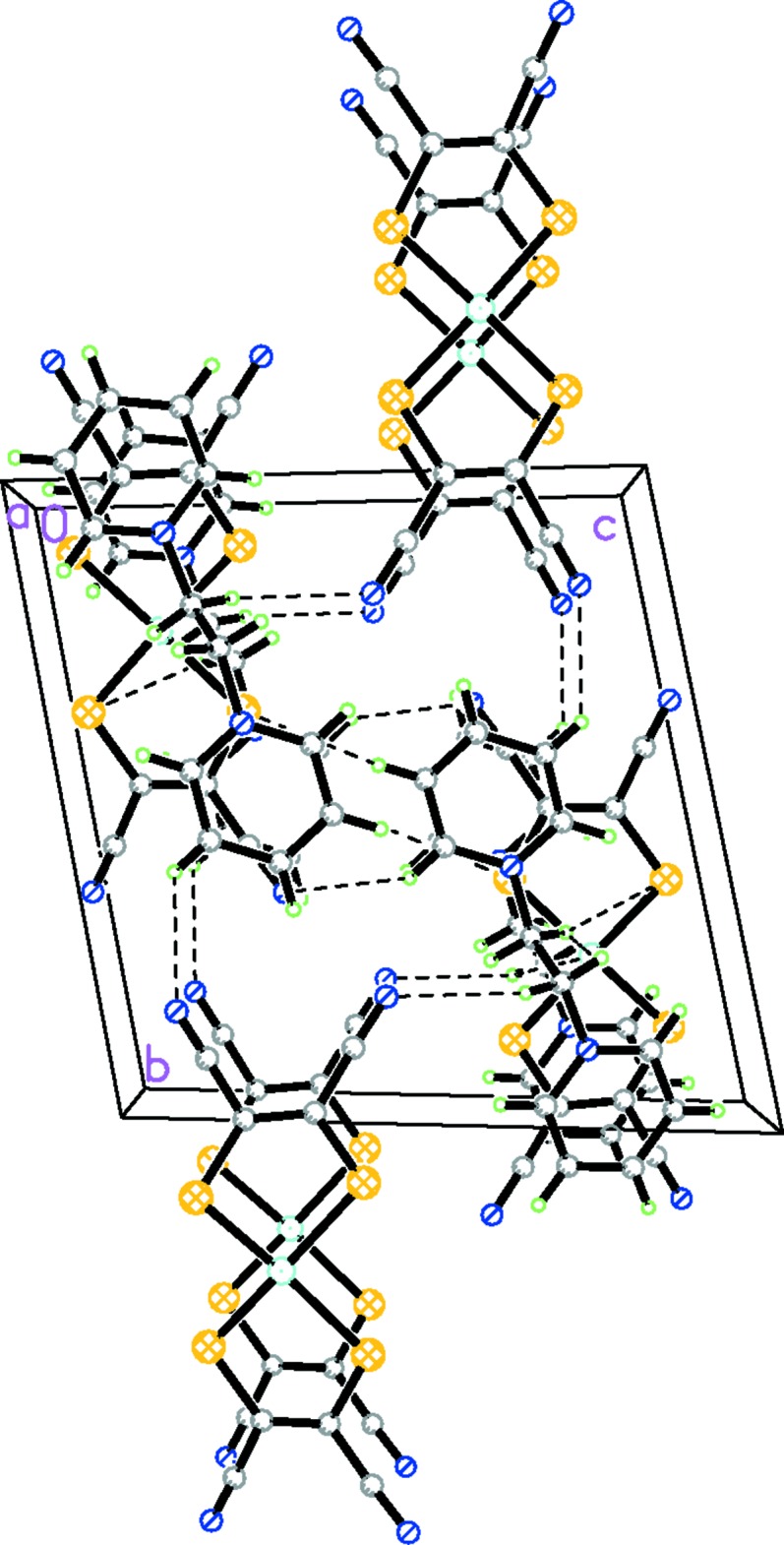
The packing diagram of the title compound as viewed along the a axis. Dashed lines denote hydrogen bonds.

## supplementary crystallographic information

### Comment

During the past few years, the molecular-based materials are widely studied due to their novel applications in the areas of materials science, medicines, biology and so on (Brammer, 2004; Robin & Fromm, 2006). Among these materials, maleonitriledithiolate (mnt) transition metal complexes are a typical kind of bis(1,2-dithiolene) complexes used as building blocks, because they possess an extended electronically delocalized core comprising a central metal, four S atoms and C═C units (Duan *et al.*, 2010; Ni *et al.*, 2005). Studies showed that weak inter- or intramolecular interactions in the complexes could influence on their properties (Ren *et al.*, 2006; Wang *et al.*, 2012).

In order to know how the cations affects the stacking mode of [Cu(mnt)_2_]^2-^ anion, herein, we present a new ion-pair complex. As shown in Fig. 1, it consists of one ethane-1,2-dipydinium (PyEtPy)^2+^ cation and a [Cu(mnt)_2_]^2-^ dianion in the asymmetric unit. The [Cu(mnt)_2_]^2-^ anion exhibits highly twisted coordination environment around the tetracoordinated Cu^II^ atom. The dihedral angle between the two mnt ligands is 37.49 (3)°, which is the largest value in this kind complexes. The dihedral angle between two pyridyl planes in the organic cation is 29.18 (10)°. It is seen from Table 1 and Fig. 2 that the crystal structure is stabilized by weak C—H···N and C—H···S hydrogen bonds, which link the cations and anions into a three-dimensional network.

### Experimental

The synthesis route of the title compound is similar to reference (Wang *et al.*, 2012). It was prepared by a direct reaction of CuCl_2_ (0.171g, 1.0 mmol), Na_2_(mnt) (0.372g, 2.0 mmol) and ethane-1,2-dipydinium bromide (0.344g, 1.0 mmol) in a mixed solution of ethanol and H_2_O (v/v 1:1; 20 ml). After filtration, brown-red block-like single crystals were obtained by slow evaporation of the crude in an acetonitrile solution at room temperature in about two weeks.

### Refinement

H atoms were positioned geometrically and refined as riding atoms, with C—H = 0.97 (methylene) and 0.93 (pyridyl) Å and with *U*_iso_(H) = 1.2*U*_eq_(C).

### Crystal data

**Table:**

(C_12_H_14_N_2_)[Cu(C_4_N_2_S_2_)_2_]	*Z* = 2
*M**_r_* = 530.20	*F*(000) = 538
Triclinic, *P*1	*D*_x_ = 1.553 Mg m^−^^3^
Hall symbol: -P 1	Mo *K*α radiation, λ = 0.71073 Å
*a* = 7.7598 (10) Å	Cell parameters from 2653 reflections
*b* = 12.3811 (15) Å	θ = 2.2–28.0°
*c* = 12.6572 (16) Å	µ = 1.35 mm^−^^1^
α = 77.676 (2)°	*T* = 291 K
β = 72.791 (2)°	Block, brown-red
γ = 84.122 (2)°	0.25 × 0.20 × 0.15 mm
*V* = 1133.8 (2) Å^3^	

### Data collection

**Table:**

Bruker APEX CCD diffractometer	3921 independent reflections
Radiation source: sealed tube	3217 reflections with *I* > 2σ(*I*)
Graphite monochromator	*R*_int_ = 0.054
φ and ω scans	θ_max_ = 25.0°, θ_min_ = 1.7°
Absorption correction: multi-scan (*SADABS*; Bruker, 2001)	*h* = −9→8
*T*_min_ = 0.730, *T*_max_ = 0.815	*k* = −14→12
5682 measured reflections	*l* = −15→15

### Refinement

**Table:**

Refinement on *F*^2^	Primary atom site location: structure-invariant direct methods
Least-squares matrix: full	Secondary atom site location: difference Fourier map
*R*[*F*^2^ > 2σ(*F*^2^)] = 0.036	Hydrogen site location: inferred from neighbouring sites
*wR*(*F*^2^) = 0.083	H-atom parameters constrained
*S* = 1.00	*w* = 1/[σ^2^(*F*_o_^2^) + (0.0333*P*)^2^] where *P* = (*F*_o_^2^ + 2*F*_c_^2^)/3
3921 reflections	(Δ/σ)_max_ < 0.001
280 parameters	Δρ_max_ = 0.39 e Å^−^^3^
1 restraint	Δρ_min_ = −0.26 e Å^−^^3^

### Special details

**Table:**

Experimental. Least-squares planes (*x*,*y*,*z* in crystal coordinates) and deviations from them (* indicates atom used to define plane)6.9701 (0.0047) *x* + 5.1850 (0.0148) *y* + 7.5933 (0.0151) *z* = 5.1170 (0.0027)* -0.0036 (0.0020) N5 * 0.0025 (0.0022) C9 * 0.0020 (0.0024) C10 * -0.0053 (0.0025) C11 * 0.0043 (0.0025) C12 * 0.0001 (0.0022) C13Rms deviation of fitted atoms = 0.00347.3331 (0.0038) *x* + 4.3436 (0.0161) *y* + 1.7102 (0.0200) *z* = 4.6388 (0.0108)Angle to previous plane (with approximate e.s.d.) = 29.18 (10)* 0.0013 (0.0022) N6 * -0.0036 (0.0025) C16 * 0.0015 (0.0028) C17 * 0.0027 (0.0028) C18 * -0.0049 (0.0027) C19 * 0.0029 (0.0024) C20Rms deviation of fitted atoms = 0.00316.9588 (0.0022) *x* + 3.8087 (0.0081) *y* - 0.7118 (0.0088) *z* = 7.0678 (0.0051)Angle to previous plane (with approximate e.s.d.) = 10.99 (12)* -0.0330 (0.0015) S1 * -0.0111 (0.0015) S2 * 0.0353 (0.0026) C1 * 0.0220 (0.0028) C4 * 0.0292 (0.0027) C2 * 0.0074 (0.0032) C3 * -0.0259 (0.0020) N1 * -0.0239 (0.0024) N2Rms deviation of fitted atoms = 0.02527.2222 (0.0021) *x* + 4.6573 (0.0073) *y* + 6.9013 (0.0071) *z* = 8.7279 (0.0018)Angle to previous plane (with approximate e.s.d.) = 36.27 (4)* 0.0321 (0.0014) S3 * 0.0067 (0.0014) S4 * -0.0293 (0.0025) C5 * -0.0251 (0.0025) C7 * 0.0012 (0.0029) C8 * -0.0242 (0.0028) C6 * 0.0188 (0.0021) N3 * 0.0197 (0.0021) N4Rms deviation of fitted atoms = 0.0220
Geometry. All e.s.d.'s (except the e.s.d. in the dihedral angle between two l.s. planes) are estimated using the full covariance matrix. The cell e.s.d.'s are taken into account individually in the estimation of e.s.d.'s in distances, angles and torsion angles; correlations between e.s.d.'s in cell parameters are only used when they are defined by crystal symmetry. An approximate (isotropic) treatment of cell e.s.d.'s is used for estimating e.s.d.'s involving l.s. planes.
Refinement. Refinement of *F*^2^ against ALL reflections. The weighted *R*-factor *wR* and goodness of fit *S* are based on *F*^2^, conventional *R*-factors *R* are based on *F*, with *F* set to zero for negative *F*^2^. The threshold expression of *F*^2^ > σ(*F*^2^) is used only for calculating *R*-factors(gt) *etc*. and is not relevant to the choice of reflections for refinement. *R*-factors based on *F*^2^ are statistically about twice as large as those based on *F*, and *R*- factors based on ALL data will be even larger.

### Fractional atomic coordinates and isotropic or equivalent isotropic displacement parameters (Å^2^)

**Table:**

	*x*	*y*	*z*	*U*_iso_*/*U*_eq_	
C1	0.7697 (4)	0.4815 (2)	0.1224 (2)	0.0391 (7)	
C2	0.7087 (4)	0.5794 (2)	0.0580 (2)	0.0438 (7)	
C3	0.7285 (5)	0.5782 (3)	0.2764 (3)	0.0534 (9)	
C4	0.7788 (4)	0.4812 (2)	0.2281 (2)	0.0399 (7)	
C5	1.0203 (4)	−0.0026 (2)	0.1945 (2)	0.0381 (7)	
C6	1.1186 (5)	−0.0989 (2)	0.1573 (2)	0.0471 (8)	
C7	0.9214 (4)	−0.0103 (2)	0.3037 (2)	0.0383 (7)	
C8	0.9143 (5)	−0.1115 (2)	0.3834 (3)	0.0476 (8)	
C9	0.4956 (4)	0.0999 (2)	0.1511 (2)	0.0473 (8)	
H9A	0.5115	0.1626	0.0943	0.057*	
C10	0.5896 (5)	0.0029 (3)	0.1309 (3)	0.0597 (10)	
H10A	0.6695	−0.0002	0.0604	0.072*	
C11	0.5661 (5)	−0.0882 (3)	0.2138 (3)	0.0638 (10)	
H11A	0.6283	−0.1545	0.2005	0.077*	
C12	0.4494 (5)	−0.0818 (3)	0.3178 (3)	0.0622 (10)	
H12A	0.4333	−0.1435	0.3759	0.075*	
C13	0.3573 (5)	0.0154 (2)	0.3354 (3)	0.0511 (8)	
H13A	0.2774	0.0200	0.4056	0.061*	
C14	0.2858 (4)	0.2108 (2)	0.2732 (3)	0.0413 (7)	
H14A	0.1753	0.1975	0.3341	0.050*	
H14B	0.2547	0.2506	0.2061	0.050*	
C15	0.4079 (4)	0.2779 (2)	0.3040 (3)	0.0453 (8)	
H15A	0.4296	0.2404	0.3747	0.054*	
H15B	0.5232	0.2838	0.2464	0.054*	
C16	0.2857 (5)	0.4217 (2)	0.4142 (3)	0.0525 (9)	
H16A	0.2996	0.3717	0.4776	0.063*	
C17	0.2222 (5)	0.5267 (3)	0.4229 (3)	0.0678 (11)	
H17A	0.1934	0.5488	0.4921	0.081*	
C18	0.2010 (5)	0.5992 (3)	0.3304 (3)	0.0662 (11)	
H18A	0.1578	0.6712	0.3359	0.079*	
C19	0.2430 (5)	0.5663 (3)	0.2295 (3)	0.0593 (10)	
H19A	0.2276	0.6150	0.1657	0.071*	
C20	0.3078 (4)	0.4609 (2)	0.2238 (3)	0.0506 (8)	
H20A	0.3384	0.4378	0.1551	0.061*	
Cu1	0.88151 (5)	0.23822 (3)	0.20268 (3)	0.03682 (13)	
N1	0.6543 (4)	0.6544 (2)	0.0053 (2)	0.0597 (8)	
N2	0.6877 (5)	0.6526 (2)	0.3190 (3)	0.0809 (11)	
N3	1.2014 (5)	−0.1729 (2)	0.1268 (2)	0.0733 (10)	
N4	0.9043 (5)	−0.1908 (2)	0.4499 (2)	0.0708 (9)	
N5	0.3814 (3)	0.10412 (17)	0.25224 (19)	0.0362 (6)	
N6	0.3283 (3)	0.39007 (18)	0.31502 (19)	0.0375 (6)	
S1	0.81723 (12)	0.36536 (6)	0.06140 (6)	0.0456 (2)	
S2	0.84617 (12)	0.36517 (6)	0.31245 (6)	0.0456 (2)	
S3	1.04488 (12)	0.11853 (6)	0.09586 (6)	0.0457 (2)	
S4	0.80239 (11)	0.10227 (6)	0.35693 (6)	0.0459 (2)	

### Atomic displacement parameters (Å^2^)

**Table:**

	*U*^11^	*U*^22^	*U*^33^	*U*^12^	*U*^13^	*U*^23^
C1	0.0419 (18)	0.0328 (15)	0.0380 (17)	0.0020 (13)	−0.0092 (14)	−0.0017 (13)
C2	0.051 (2)	0.0408 (17)	0.0368 (17)	−0.0010 (15)	−0.0112 (15)	−0.0036 (14)
C3	0.079 (3)	0.0398 (18)	0.049 (2)	0.0081 (17)	−0.0337 (19)	−0.0061 (16)
C4	0.0478 (19)	0.0299 (15)	0.0422 (18)	0.0034 (13)	−0.0154 (15)	−0.0060 (13)
C5	0.0471 (19)	0.0306 (15)	0.0361 (17)	0.0025 (13)	−0.0127 (14)	−0.0060 (12)
C6	0.067 (2)	0.0352 (17)	0.0350 (17)	0.0019 (15)	−0.0125 (16)	−0.0032 (14)
C7	0.0486 (19)	0.0275 (14)	0.0375 (17)	−0.0017 (13)	−0.0135 (14)	−0.0018 (12)
C8	0.063 (2)	0.0393 (18)	0.0370 (18)	−0.0026 (15)	−0.0067 (16)	−0.0102 (15)
C9	0.063 (2)	0.0375 (17)	0.0339 (17)	0.0012 (15)	−0.0050 (16)	−0.0033 (13)
C10	0.065 (2)	0.054 (2)	0.050 (2)	0.0092 (18)	0.0008 (18)	−0.0185 (18)
C11	0.076 (3)	0.0374 (19)	0.082 (3)	0.0137 (18)	−0.026 (2)	−0.0213 (19)
C12	0.090 (3)	0.0356 (18)	0.059 (2)	0.0010 (18)	−0.027 (2)	0.0027 (16)
C13	0.069 (2)	0.0411 (18)	0.0373 (18)	−0.0007 (16)	−0.0114 (17)	−0.0022 (14)
C14	0.0418 (18)	0.0342 (15)	0.0461 (18)	0.0081 (13)	−0.0112 (15)	−0.0103 (13)
C15	0.0436 (19)	0.0362 (16)	0.056 (2)	0.0069 (14)	−0.0153 (16)	−0.0110 (14)
C16	0.079 (3)	0.0449 (19)	0.0355 (18)	−0.0065 (17)	−0.0199 (17)	−0.0051 (14)
C17	0.108 (3)	0.050 (2)	0.042 (2)	0.002 (2)	−0.010 (2)	−0.0206 (17)
C18	0.093 (3)	0.0376 (19)	0.065 (3)	0.0082 (19)	−0.017 (2)	−0.0171 (18)
C19	0.086 (3)	0.0396 (19)	0.052 (2)	0.0044 (18)	−0.026 (2)	−0.0020 (16)
C20	0.071 (2)	0.0458 (18)	0.0339 (18)	−0.0015 (16)	−0.0114 (16)	−0.0099 (14)
Cu1	0.0427 (2)	0.0313 (2)	0.0346 (2)	0.00331 (15)	−0.01034 (17)	−0.00546 (15)
N1	0.071 (2)	0.0499 (17)	0.0505 (18)	0.0040 (15)	−0.0195 (16)	0.0059 (14)
N2	0.139 (3)	0.0435 (18)	0.074 (2)	0.0230 (19)	−0.053 (2)	−0.0209 (17)
N3	0.108 (3)	0.0440 (17)	0.058 (2)	0.0173 (17)	−0.0132 (19)	−0.0122 (15)
N4	0.108 (3)	0.0420 (17)	0.0482 (18)	−0.0037 (17)	−0.0094 (18)	0.0032 (15)
N5	0.0413 (15)	0.0309 (12)	0.0347 (14)	0.0027 (11)	−0.0111 (12)	−0.0043 (10)
N6	0.0417 (15)	0.0319 (13)	0.0377 (14)	0.0017 (11)	−0.0096 (12)	−0.0075 (11)
S1	0.0638 (6)	0.0395 (4)	0.0309 (4)	0.0057 (4)	−0.0127 (4)	−0.0057 (3)
S2	0.0669 (6)	0.0341 (4)	0.0418 (5)	0.0090 (4)	−0.0272 (4)	−0.0091 (3)
S3	0.0639 (6)	0.0335 (4)	0.0312 (4)	0.0070 (4)	−0.0053 (4)	−0.0034 (3)
S4	0.0571 (5)	0.0370 (4)	0.0342 (4)	0.0031 (4)	−0.0018 (4)	−0.0046 (3)

### Geometric parameters (Å, º)

**Table:**

C1—C4	1.360 (4)	C13—H13A	0.9300
C1—C2	1.431 (4)	C14—N5	1.482 (3)
C1—S1	1.730 (3)	C14—C15	1.503 (4)
C2—N1	1.143 (3)	C14—H14A	0.9700
C3—N2	1.136 (4)	C14—H14B	0.9700
C3—C4	1.430 (4)	C15—N6	1.479 (3)
C4—S2	1.732 (3)	C15—H15A	0.9700
C5—C7	1.357 (4)	C15—H15B	0.9700
C5—C6	1.437 (4)	C16—N6	1.333 (3)
C5—S3	1.721 (3)	C16—C17	1.357 (4)
C6—N3	1.134 (3)	C16—H16A	0.9300
C7—C8	1.425 (4)	C17—C18	1.356 (4)
C7—S4	1.738 (3)	C17—H17A	0.9300
C8—N4	1.141 (4)	C18—C19	1.361 (4)
C9—N5	1.332 (4)	C18—H18A	0.9300
C9—C10	1.371 (4)	C19—C20	1.358 (4)
C9—H9A	0.9300	C19—H19A	0.9300
C10—C11	1.353 (5)	C20—N6	1.332 (4)
C10—H10A	0.9300	C20—H20A	0.9300
C11—C12	1.373 (5)	Cu1—S3	2.2554 (8)
C11—H11A	0.9300	Cu1—S1	2.2561 (8)
C12—C13	1.361 (4)	Cu1—S2	2.2571 (8)
C12—H12A	0.9300	Cu1—S4	2.2630 (8)
C13—N5	1.335 (4)		
			
C4—C1—C2	120.1 (2)	N6—C15—C14	111.5 (2)
C4—C1—S1	123.3 (2)	N6—C15—H15A	109.3
C2—C1—S1	116.5 (2)	C14—C15—H15A	109.3
N1—C2—C1	176.5 (3)	N6—C15—H15B	109.3
N2—C3—C4	177.2 (3)	C14—C15—H15B	109.3
C1—C4—C3	120.8 (2)	H15A—C15—H15B	108.0
C1—C4—S2	122.9 (2)	N6—C16—C17	120.2 (3)
C3—C4—S2	116.3 (2)	N6—C16—H16A	119.9
C7—C5—C6	119.5 (2)	C17—C16—H16A	119.9
C7—C5—S3	124.0 (2)	C18—C17—C16	119.7 (3)
C6—C5—S3	116.5 (2)	C18—C17—H17A	120.1
N3—C6—C5	177.7 (4)	C16—C17—H17A	120.1
C5—C7—C8	121.6 (2)	C17—C18—C19	119.9 (3)
C5—C7—S4	122.7 (2)	C17—C18—H18A	120.1
C8—C7—S4	115.7 (2)	C19—C18—H18A	120.1
N4—C8—C7	177.6 (3)	C20—C19—C18	118.7 (3)
N5—C9—C10	119.9 (3)	C20—C19—H19A	120.6
N5—C9—H9A	120.0	C18—C19—H19A	120.6
C10—C9—H9A	120.0	N6—C20—C19	121.1 (3)
C11—C10—C9	119.9 (3)	N6—C20—H20A	119.4
C11—C10—H10A	120.1	C19—C20—H20A	119.4
C9—C10—H10A	120.1	S3—Cu1—S1	97.08 (3)
C10—C11—C12	119.3 (3)	S3—Cu1—S2	153.90 (4)
C10—C11—H11A	120.4	S1—Cu1—S2	92.15 (3)
C12—C11—H11A	120.4	S3—Cu1—S4	92.28 (3)
C13—C12—C11	119.6 (3)	S1—Cu1—S4	152.11 (4)
C13—C12—H12A	120.2	S2—Cu1—S4	90.80 (3)
C11—C12—H12A	120.2	C9—N5—C13	121.1 (2)
N5—C13—C12	120.2 (3)	C9—N5—C14	119.0 (2)
N5—C13—H13A	119.9	C13—N5—C14	119.9 (2)
C12—C13—H13A	119.9	C20—N6—C16	120.3 (2)
N5—C14—C15	108.6 (2)	C20—N6—C15	119.3 (2)
N5—C14—H14A	110.0	C16—N6—C15	120.2 (2)
C15—C14—H14A	110.0	C1—S1—Cu1	100.72 (10)
N5—C14—H14B	110.0	C4—S2—Cu1	100.81 (9)
C15—C14—H14B	110.0	C5—S3—Cu1	100.54 (9)
H14A—C14—H14B	108.4	C7—S4—Cu1	100.40 (10)
			
C2—C1—C4—C3	−0.3 (5)	C17—C16—N6—C20	−0.4 (5)
S1—C1—C4—C3	−176.6 (3)	C17—C16—N6—C15	176.2 (3)
C2—C1—C4—S2	178.5 (2)	C14—C15—N6—C20	−67.1 (4)
S1—C1—C4—S2	2.1 (4)	C14—C15—N6—C16	116.2 (3)
C6—C5—C7—C8	0.8 (5)	C4—C1—S1—Cu1	1.0 (3)
S3—C5—C7—C8	−176.3 (2)	C2—C1—S1—Cu1	−175.5 (2)
C6—C5—C7—S4	178.5 (2)	S3—Cu1—S1—C1	−158.09 (11)
S3—C5—C7—S4	1.4 (4)	S2—Cu1—S1—C1	−2.55 (11)
N5—C9—C10—C11	0.0 (5)	S4—Cu1—S1—C1	93.23 (12)
C9—C10—C11—C12	−0.8 (5)	C1—C4—S2—Cu1	−3.9 (3)
C10—C11—C12—C13	1.0 (6)	C3—C4—S2—Cu1	174.9 (2)
C11—C12—C13—N5	−0.5 (5)	S3—Cu1—S2—C4	114.23 (12)
N5—C14—C15—N6	174.3 (2)	S1—Cu1—S2—C4	3.32 (11)
N6—C16—C17—C18	0.4 (6)	S4—Cu1—S2—C4	−148.94 (11)
C16—C17—C18—C19	0.2 (6)	C7—C5—S3—Cu1	1.0 (3)
C17—C18—C19—C20	−0.8 (6)	C6—C5—S3—Cu1	−176.2 (2)
C18—C19—C20—N6	0.8 (6)	S1—Cu1—S3—C5	−155.81 (11)
C10—C9—N5—C13	0.5 (5)	S2—Cu1—S3—C5	94.35 (12)
C10—C9—N5—C14	178.0 (3)	S4—Cu1—S3—C5	−2.14 (11)
C12—C13—N5—C9	−0.3 (5)	C5—C7—S4—Cu1	−2.9 (3)
C12—C13—N5—C14	−177.7 (3)	C8—C7—S4—Cu1	174.9 (2)
C15—C14—N5—C9	−83.5 (3)	S3—Cu1—S4—C7	2.65 (11)
C15—C14—N5—C13	94.0 (3)	S1—Cu1—S4—C7	112.46 (11)
C19—C20—N6—C16	−0.2 (5)	S2—Cu1—S4—C7	−151.43 (10)
C19—C20—N6—C15	−176.9 (3)		

### Hydrogen-bond geometry (Å, º)

**Table:**

*D*—H···*A*	*D*—H	H···*A*	*D*···*A*	*D*—H···*A*
C11—H11*A*···N2^i^	0.93	2.61	3.356 (4)	137
C14—H14*A*···N4^ii^	0.97	2.60	3.341 (4)	133
C14—H14*B*···N1^iii^	0.97	2.60	3.479 (4)	151
C15—H15*B*···S1	0.97	2.83	3.769 (3)	163
C16—H16*A*···N2^iv^	0.93	2.56	3.368 (4)	146
C17—H17*A*···S2^iv^	0.93	2.82	3.743 (3)	171
C19—H19*A*···N3^v^	0.93	2.56	3.235 (4)	129
C20—H20*A*···N1^iii^	0.93	2.52	3.422 (4)	164

Symmetry codes: (i) *x*, *y*−1, *z*; (ii) −*x*+1, −*y*, −*z*+1; (iii) −*x*+1, −*y*+1, −*z*; (iv) −*x*+1, −*y*+1, −*z*+1; (v) *x*−1, *y*+1, *z*.
